# Effect of Graphic Warning Labels on Cigarette Packs on US Smokers’ Cognitions and Smoking Behavior After 3 Months: A Randomized Clinical Trial

**DOI:** 10.1001/jamanetworkopen.2021.21387

**Published:** 2021-08-04

**Authors:** David R. Strong, John P. Pierce, Kim Pulvers, Matthew D. Stone, Adriana Villaseñor, Minya Pu, Claudiu V. Dimofte, Eric C. Leas, Jesica Oratowski, Elizabeth Brighton, Samantha Hurst, Sheila Kealey, Ruifeng Chen, Karen Messer

**Affiliations:** 1 Cancer Control Program, Moores Cancer Center, University of California, San Diego, La Jolla; 2 Herbert Wertheim School of Public Health and Human Longevity Science, University of California, San Diego, La Jolla; 3 Department of Psychology, California State University San Marcos, San Marcos; 4 Department of Epidemiology, Public Health Services, San Diego County, San Diego, California; 5 Department of Marketing, San Diego State University, San Diego, California

## Abstract

**Importance:**

The US Food and Drug Administration’s implementation of graphic warning labels (GWLs) on cigarette packs is under challenge in US courts.

**Objective:**

To determine whether GWLs can affect US smokers’ perceptions about their cigarettes or health consequences and changes in smoking behavior.

**Design, Setting, and Participants:**

This study was a randomized clinical trial of the effect of a 3-month, real-world experience of cigarettes with GWL packaging. Community recruitment was done from September 2016 through December 2019 of daily smokers from San Diego, California, aged 21 to 65 years, who were not ready to quit. Participants were randomized to purchase and receive cigarettes in 1 of 3 pack designs: GWL, blank, or standard US pack. Data analysis was performed from July 2020 to February 2021.

**Interventions:**

The study manufactured GWL cigarette packs (3 versions with Australian-licensed images) and packs devoid of marketing. For 3 months, participants purchased GWL, blank, or standard US pack cigarettes that were delivered to their home.

**Main Outcomes and Measures:**

Smoking-related cognitions and behavior were queried by daily and weekly interactive text messages. Smoking behavior was self-reported before and after the intervention by 96% of randomized participants and was biochemically validated on a subsample.

**Results:**

The study sample included 357 participants (195 women [54.6%]; mean [SD] age, 39.5 [11.9] years); 116 were randomized to the standard US pack group, 118 were randomized to the GWL pack group, and 125 were randomized to the blank pack group. Over the 3 months, participants who received the GWL packs had reduced positive perceptions of recent cigarettes smoked compared with participants who received the branded US pack (mean difference, −0.46 SD; 95% CI, −0.73 SD to −0.20 SD; *P* < .001). Health concerns increased in all groups, with a significant increase in the GWL group vs the US pack group (mean difference, 0.35 SD; 95% CI, 0.09 SD to 0.62 SD; *P* = .002). Quitting cognitions increased in all study groups, with a peak mean change of 0.60 SD for GWL participants vs 0.34 SD for US pack participants (mean difference, 0.55 SD; 95% CI, 0.28 SD to 0.81 SD; *P* < .001). GWL participants had slightly more cigarette abstinence periods per week than the US pack group, but the difference was not significant (adjusted odds ratio, 1.06; 95% CI, 0.99 to 1.13). At 3 months, there was no between-group difference in any smoking behavior. The blank pack group was similar to the US pack group on all measures.

**Conclusions and Relevance:**

These findings suggest that the introduction of GWL packs appears to decrease positive perceptions of cigarettes and increase quitting cognitions in the short term. However, additional complementary tobacco control strategies may be necessary for GWL packs to be associated with reduced smoking behavior.

**Trial Registration:**

ClinicalTrials.gov Identifier: NCT02676193

## Key Points

**Question:**

Can graphic warning labels on cigarette packs affect cognitions and smoking behavior among US daily smokers who are not ready to quit smoking?

**Findings:**

This randomized clinical trial found that graphic warning labels decreased positive perceptions of cigarettes associated with branded cigarette packs but without clearly increasing health concerns. They also increased quitting cognitions but did not affect either cigarette cessation or consumption levels.

**Meaning:**

Placing graphic warning labels on US cigarette packs did not have an effect on smoking behavior; however, these findings suggest that they may enhance other tobacco control strategies to reduce cigarette smoking.

## Teaser

This randomized clinical trial examines whether graphic warning labels on cigarette packs can affect US smokers’ perceptions about their cigarettes or health consequences and changes in smoking behavior.

## Introduction

Graphic warning labels (GWLs) on tobacco packages are recommended as a cost-effective means to increase public awareness about the dangers of tobacco at the time of use.^1^ When combined with other strategies, such as smoking cessation assistance, they can provide a way for governments to reduce the health costs associated with cigarette smoking.^2^ GWLs on cigarette packs were mandated by the US Congress in 2009. However, the US Food and Drug Administration’s planned implementation, first in 2012 and again in 2020,^3^ was challenged by tobacco industry lawsuits, and the issue is still before the courts.^4^ Smokers usually have their cigarette pack in easy reach, thus providing a unique opportunity to reinforce marketing messages at the time a person considers smoking.^5^ Industry marketing research^6^ and peer-reviewed publications^7,8,9,10,11^ show that package imagery can influence smokers’ ratings of cigarette taste and satisfaction, as well as their cognitions regarding cigarettes’ safety compared with alternatives.^11^

The health and societal costs that follow a long-term cigarette addiction provide governments with a vested interest to restrict or counter marketing that promotes cigarette smoking, leading to bans or limits on cigarette marketing in the US and elsewhere.^12^ More than 120 countries have mandated that sections of the cigarette pack feature GWLs depicting the health consequences of smoking.^13^ Seven countries, including Australia, the United Kingdom, and France, have required the removal of all industry branding to eliminate any marketing that might encourage smoking.^14^ Typically, these countries have also required larger GWLs than those proposed for US packs.

Earlier US trials^15^ identified that GWLs that produced negative affect in smokers resulted in higher risk perceptions and quit intentions. There is also evidence of increased quit attempts when smokers had a GWL affixed to their cigarette pack.^16^ However, because GWLs also force a reduction in brand imagery on packs, there is a need for research to identify whether displacing branding or adding GWLs to packaging is associated with changes in smoking cognitions and behavior. Two studies have investigated whether GWLs are associated with changes in smoking behavior: a time-series analysis of Canadian data reported that GWLs were associated with reduced smoking behavior,^17^ whereas an analysis of similar data from 60 countries found no such association.^18^

This article presents the results of a randomized clinical trial examining a real-world experience with different cigarette packaging among US smokers.^19^ Participants were randomized to a study-manufactured blank pack, a pack with a GWL, or their usual US pack and purchased these cigarettes from a study website for 3 months. We previously demonstrated among these participants that such repackaging was associated with markedly different affective reactions.^19,20^ This study’s 3 aims investigate between-group differences in smokers’ concern about health risks (assessed weekly), positive perceptions about their cigarettes (assessed daily), and changes in consumption and smoking status before and after the intervention.

## Methods

From September 2016 through December 2019, we recruited daily smokers (daily smoking was defined as ≥5 cigarettes per day) aged 21 to 65 years who were not ready to quit using community and social media advertising. Participants were from San Diego, California, and signed an informed consent form approved by the institutional review boards of University of California San Diego and California State University San Marcos, both of which also approved this study. The trial protocol (Supplement 1) and analytical plans have been published elsewhere.^19^ This report follows the Consolidated Standards of Reporting Trials (CONSORT) reporting guideline.^21^ See eFigure 1 in Supplement 2 for the study design.

### Study Packs

We manufactured 4 types of study cigarette packs.^19^ Three of these included GWLs currently in use and licensed from the Commonwealth of Australia^22^—foot gangrene, neonatal baby, and throat cancer (eFigure 2 in Supplement 2)—images that have been demonstrated to elicit a range of negative affect^19^ thought necessary to counter the social cues and brand imagery of cigarettes.^23^ We also manufactured blank packs, devoid of all marketing but with the US Surgeon General’s warning. The base color for all packs was an unappealing olive color.

### Participants

**Figure 1.**
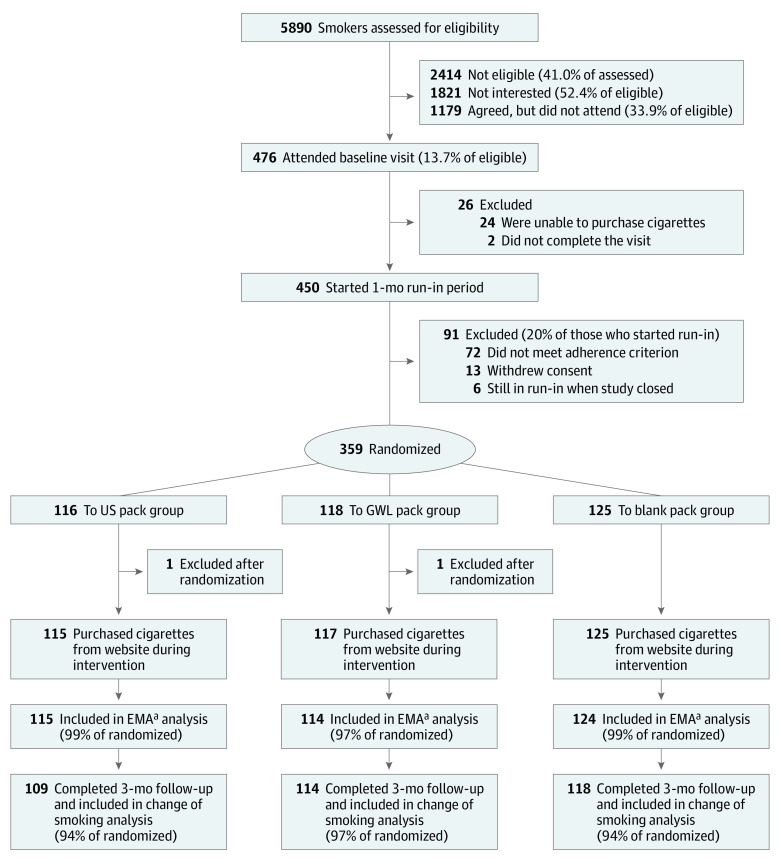
CONSORT Diagram for California Smokers in Australia (CASA) Study 3-Month Intervention GWL indicates graphic warning label. ^a^EMA refers to ecological momentary assessment by interactive daily texting to assess short-term smoking behavior and cognition.

The enrollment target was 150 participants per group to give power greater than 0.85 (α = .025) for medium between-group effects (Cohen *d* ≥ 0.25) with dropout at 20%. Community recruitment methods elicited contacts from 5890 smokers, although only 476 smokers (13.7% of eligible smokers) attended the baseline visit (visit 1), with most of those eligible either declining or not attending visit 1 (Figure 1). A further 26 participants were excluded (2 did not complete visit 1, and 24 were unable to purchase cigarettes), and 450 participants started a 1-month run-in period. There were 359 participants who adhered to the study protocol. After stratifying them on 3 variables with 2 levels each—age (<45 or ≥45 years old), sex (male or female), and nicotine dependence scores^24^ (low-to-moderate dependence or high dependence)—we used an urn randomization method^25^ to allocate participants to the study’s 3 groups (US pack, GWL pack, or blank pack). Two postrandomization exclusions (participants in different study groups were cohabitating) resulted in a study population of 357. One participant from each of the GWL and blank pack groups did not purchase any study cigarettes and, hence, did not receive the intervention. Of those randomized, 115 participants in the US pack group (99%), 114 participants in the GWL pack group (97%), and 124 participants in the blank pack group (99%) completed some daily interactive texting and were included in the ecological momentary assessment (EMA) analysis by interactive daily texting to assess short-term smoking behavior and cognitions. Long-term (3-month) smoking behavior change was assessed at the 3-month visit (visit 2) that was completed by 343 participants (96% of those randomized).

As previously published,^19^ the study achieved good comparability across groups on sociodemographic variables and factors associated with quitting (eTable 1 in Supplement 2). There were no between-group differences in terms of sex, race/ethnicity, education, or income levels. Self-reported race and ethnicity were assessed in this study to capture physical and cultural identity as mandated by the National Institutes of Health. There was no between-group difference in baseline smoking variables (cigarettes per day, recent quit attempts, nicotine dependence, current cigarette brand, loyalty to current brand, and appeal of current pack).

### Daily Assessments

EMAs delivered randomly twice daily (once early in the day and once late in the day) allowed interactive texting to capture real-time measures of smoking cognitions and behavior. Positive perceptions of cigarettes were assessed with 4-point Likert scale responses (strongly disagree, disagree, agree, and strongly agree) to these statements: “My last cigarette was satisfying,” “I enjoyed the taste of my last cigarette,” and “My last cigarette relieved my craving.” Smoking behavior was assessed with this question: “In the last 4 hours, how many cigarettes did you smoke?” We documented the proportion of participants with at least 1 weekly 4-hour period of cigarette abstinence.

### Weekly Survey

Weekly interactive texting requested responses using a 4-point Likert scale (never, some of the time, most of the time, or always) to 2 health perception questions—“How often did you think about the effect of smoking on your health?” and “How often did you think about the effects of smoking on others?”—as well as a quitting cognitions question, “How often did you think about wanting to quit?” Early technical difficulties resulted in missing data for 24 participants (6.7%) for this weekly survey.

### Study Visits and Saliva Cotinine

At both study visits, all participants completed a detailed tobacco use questionnaire that included the questions, “On how many of the past 30 days did you smoke cigarettes?” and “During the past 30 days, on the days that you did smoke, about how many cigarettes did you usually smoke per day?” Participants provided a saliva sample that was stored for later analysis.^26^ Saliva collection was suspended because of COVID-19; overall, 240 participants (67%) had a baseline and 3-month sample that were analyzed in duplicate within the same analytic batch using an enzyme-linked immunosorbent assay kit (Salimetrics USA).^27^

### Statistical Analysis

We aggregated the texting data to provide a normalized mean daily score (daily mean of participant’s observed scores minus participant’s mean score for the run-in period) across the 3 questions that made up a positive perceptions scale (satisfaction, taste, and craving relief) and the 2 questions on health concerns. To compare patterns across study groups, we used a generalized linear or linear mixed-effect model with compound symmetry covariance structure for each outcome of interest. The mean was modeled as a linear trend over time, with 0 slope for each group in the run-in period and arbitrary slope (group times slope interaction) for each group in the intervention period. For the intervention period for each group, we also allowed a knot at 60 days (to allow for change in slope during the third intervention month) if supported by a likelihood ratio test. We used a Wald test for statistical inference regarding the model-fitted mean difference in intervention effects between any 2 study groups and for differences between slopes for any 2 study groups. Mean differences are presented normalized to the SD of the measure (mean difference of a measure divided by the SD of the measure) for comparability. For binary outcomes (such as the proportion with at least 1 weekly 4-hour period of smoking abstinence), the logit link function was used to compare the odds of reporting at least 1 abstinence period between groups, with adjustment for abstinence level during the run-in phase. For cigarette consumption and other outcomes, a linear regression model (identity link) was used. All models were adjusted for age group (<45 vs ≥45 years), sex, and baseline nicotine dependence levels. We used R statistical software version 4.0.4 (R Project for Statistical Computing).^28^ All tests were 2-sided and used a priori significance of *P* < .05. Data analysis was performed from July 2020 to February 2021.

## Results

The study sample included 357 participants (mean [SD] age, 39.5 [11.9] years); 116 were randomized to the US pack group, 118 were randomized to the GWL pack group, and 125 were randomized to the blank pack group. There were no between-group differences in terms of sex (195 women [54.6%]), race/ethnicity (245 White participants [68.2%], 40 Hispanic participants [11.0%], and 74 other non-Hispanic participants [20.0%]), education (149 participants with a college degree [41.5%], 168 participants with some college [46.8%], and 42 participants with a high school education or less [11.7%]), or income levels (143 participants [39.8%] earned <$50 000 per year) (eTable 1 in Supplement 2).

### Adherence With Study Protocol

We delivered 18 987 cigarette packs during the 4 months (1-month run-in plus 3-month intervention). A mean of 41.9 (95% CI, 38.8-45.0) packs were delivered to the US pack group, 38.4 (95% CI, 35.2-41.6) packs were delivered to the GWL pack group, and 35.3 (95% CI, 32.4-38.2) packs were delivered to the blank pack group. From self-reported consumption at visit 1 and visit 2, we estimate that approximately 60% of those who received study manufactured packs received more than one-half of the cigarettes that they smoked from study packs. The mean (SD) participant response to the daily EMA texts was similar among groups over the 3 months: 91 (47) text responses for the US pack group, 85 (42) text responses for the GWL pack group, and 88 (40) text responses for the blank pack group.

### Change in Positive Perceptions of Cigarettes

**Figure 2.**
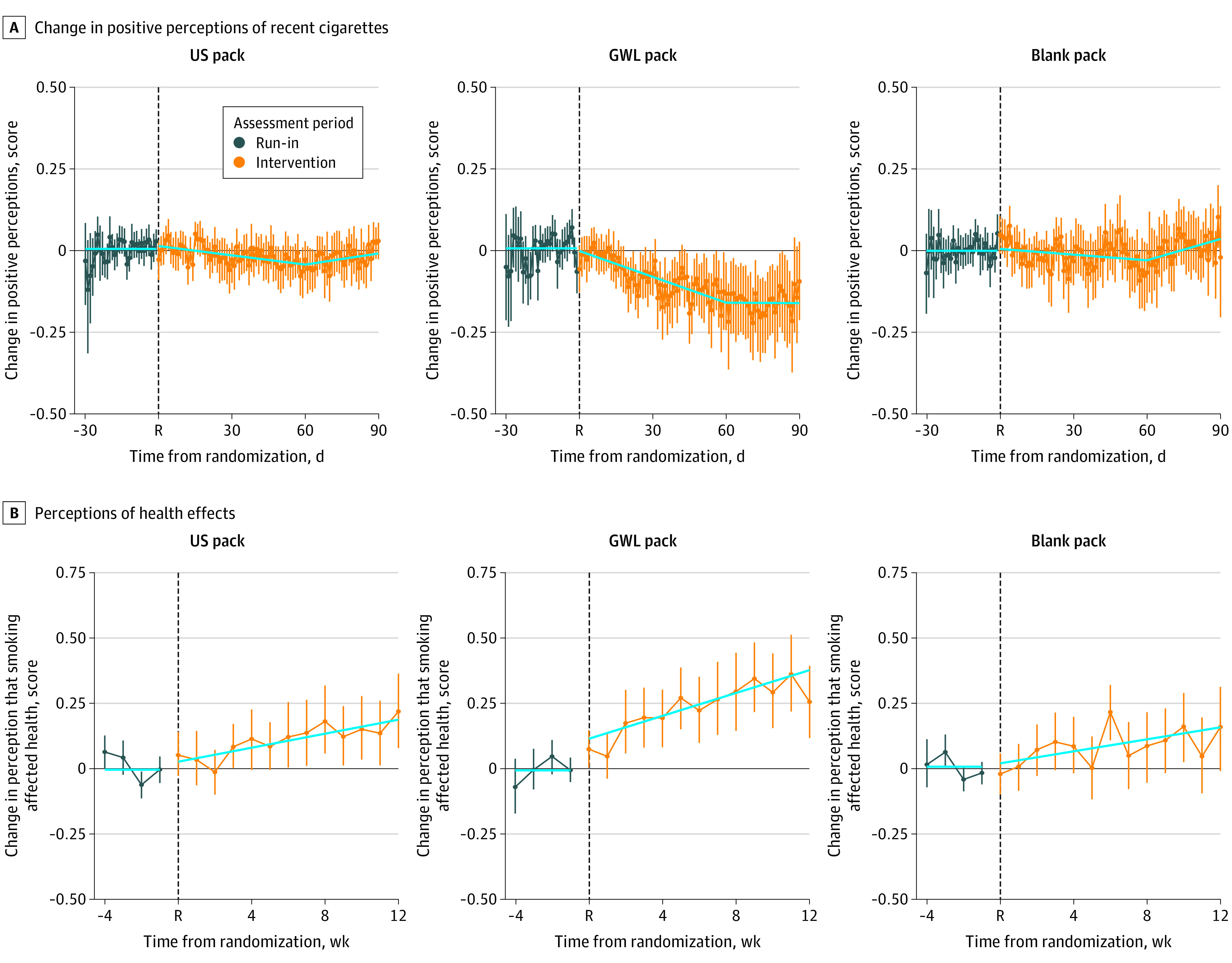
Change in Positive Perceptions of Recent Cigarettes and Perceptions of Health Effects Reported During Ecological Momentary Assessments (EMAs) by Interactive Texting A, Positive perceptions of recent cigarettes are rated on a 4-point scale (1 = strongly disagree; 2 = disagree; 3 = agree; 4 = strongly agree) formed from 3 questions on satisfaction, craving relief, and taste of recent cigarettes assessed during daily EMAs throughout the study. Data were available for 111 participants in the US pack group, 112 participants in the graphic warning label (GWL) pack group, and 123 participants in the blank pack group and were normalized to reflect differences from an average rating during the 1-month baseline period. B, The Health Perception Scale is a 4-point scale formed from 2 questions on how the participant’s smoking impacted their health and that of others, assessed on weekly EMAs. Data were available for 109 US pack participants, 109 GWL pack participants, and 113 blank pack participants and were normalized to reflect differences from a mean rating during the 1-month baseline period. During the run-in period, all groups received the US pack. At week 0 (randomization), participants were allocated to a study pack group. The solid line denotes the mean predicted values from a mixed-effects model with compound symmetry covariance structure; run-in slope was set to 0; randomization period slope incorporates a knot at 60 days (panel A). Vertical segments are observed mean changes in scores and their 95% CIs. Details are presented in eTable 2 and eTable 3 in Supplement 2.

Participants’ positive cigarette perceptions formed a reliable 4-point scale (1, strongly disagree; 2, disagree; 3, agree; 4, strongly agree; Cronbach α, 0.87), and the mean (SD) rating during run-in was 3.00 (0.49) for agree. Intervention changes in this scale were best fit by a model with a knot at 60 days (Figure 2A). There was no change in positive perceptions among smokers in either the US or blank pack groups (eTable 2 in Supplement 2). Compared with the US pack group, the GWL pack group experienced a consistently faster decrease in these positive perceptions over the first 2 months of the intervention (mean difference, −0.46 SD; 95% CI, −0.73 SD to −0.20 SD; *P* < .001) so that at the end of the intervention, they were almost half a SD below the US pack group (–0.07; 95% CI, −0.03 to −0.12).

The 2 questions on perceptions of health concerns were correlated and made a reliable 4-point scale (1, never; 2, some of the time; 3, most of the time; 4, always) (*r* = 0.49; scalability coefficient *H* = 0.54) and were normalized to reflect changes from the mean rating during the run-in period when participants thought of health effects some of the time (mean [SD], 1.97 [0.57]). Health concerns increased in all 3 groups during the intervention (Figure 2B), with the GWL group increasing more than the US pack group (mean difference, 0.35 SD; 95% CI, 0.09 SD to 0.62 SD; *P* = .002; standardized effect, 0.12; 95% CI, 0.03 to 0.21) (see eTable 3 in Supplement 2 for raw units).

### Change in Quitting Cognitions and Abstinence Periods

**Figure 3.**
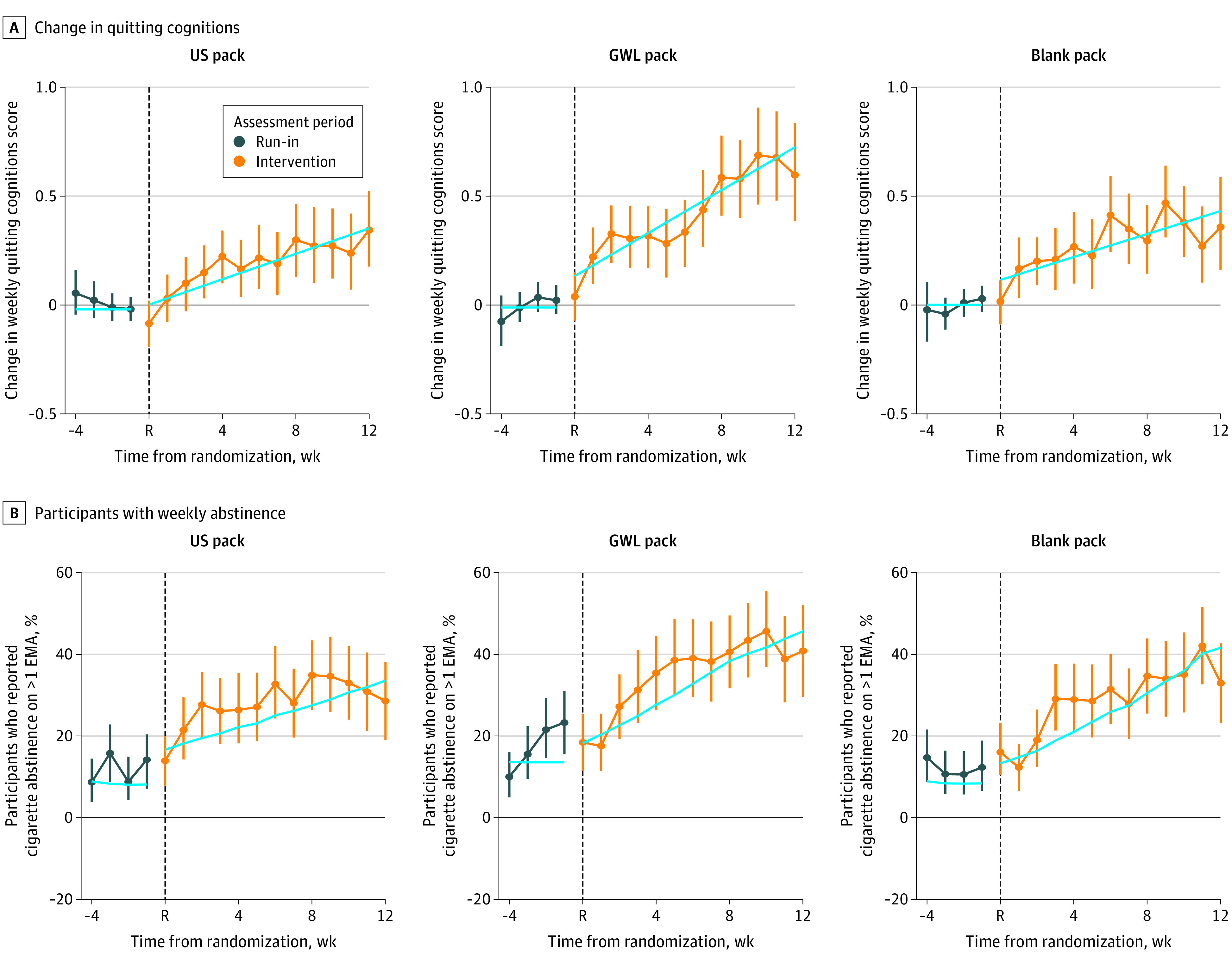
Change in Quitting Cognitions and Abstinence Periods Reported During Weekly Ecological Momentary Assessments (EMAs)by Interactive Texting Quitting cognitions were assessed on a 4-point scale during weekly EMAs. Weekly abstinence was the proportion of participants with at least 1 daily 4-hour interactive text period in which no smoking was reported. For both variables, data were available for 109 US pack group participants, 109 graphic warning label (GWL) pack group participants, and 113 blank pack group participants. During the run-in period, all groups received the US pack, and at week 0 (randomization [R]), participants were allocated to a study pack group for the 12-week intervention. The solid line denotes the mean predicted values from a generalized linear mixed model (panel A, normal; panel B, logistic) with compound symmetry covariance structure; run-in slope set to 0. Vertical segments are observed mean changes in scores (A) or proportions (B) and their 95% CIs. Details are presented in eTable 4 and eTable 5 in Supplement 2.

Quitting cognitions (4-point scale: 1, never; 2, some of the time; 3, most of the time; 4, always) were normalized to mean baseline values (mean [SD], 1.85 [0.65]), indicating some of the time, and increased in all groups over the 3-month intervention (Figure 3A). As shown in eTable 4 in Supplement 2, there was no difference in the pattern of increase in cognitions between the US and blank pack groups, although the increase was greater in the GWL pack group (peak mean change of 0.60 for GWL participants vs 0.34 for US pack participants; mean difference, 0.55 SD; 95% CI, 0.28 SD to 0.81 SD; *P* < .001; GWL vs US standardized difference, −0.23; 95% CI, 0.34 to 0.12).

We report trends in the number of participants who reported at least 1 weekly 4-hour abstinence period. Over the 3-month intervention, such abstinences increased in all 3 study groups (Figure 3B). The weekly proportion with such a cigarette abstinence period peaked at 35% (41 participants) in the US pack group, at 40% (50 participants) in the blank pack group, and at 45% (52 participants) in the GWL group. As shown in eTable 5 in Supplement 2, the GWL pack group experienced a slightly higher proportion of participants with such a weekly abstinence than the US pack group, although the difference was not significant (adjusted odds ratio, 1.06; 95% CI, 0.99-1.13).

### Change in Daily Cigarette Consumption

**Figure 4.**
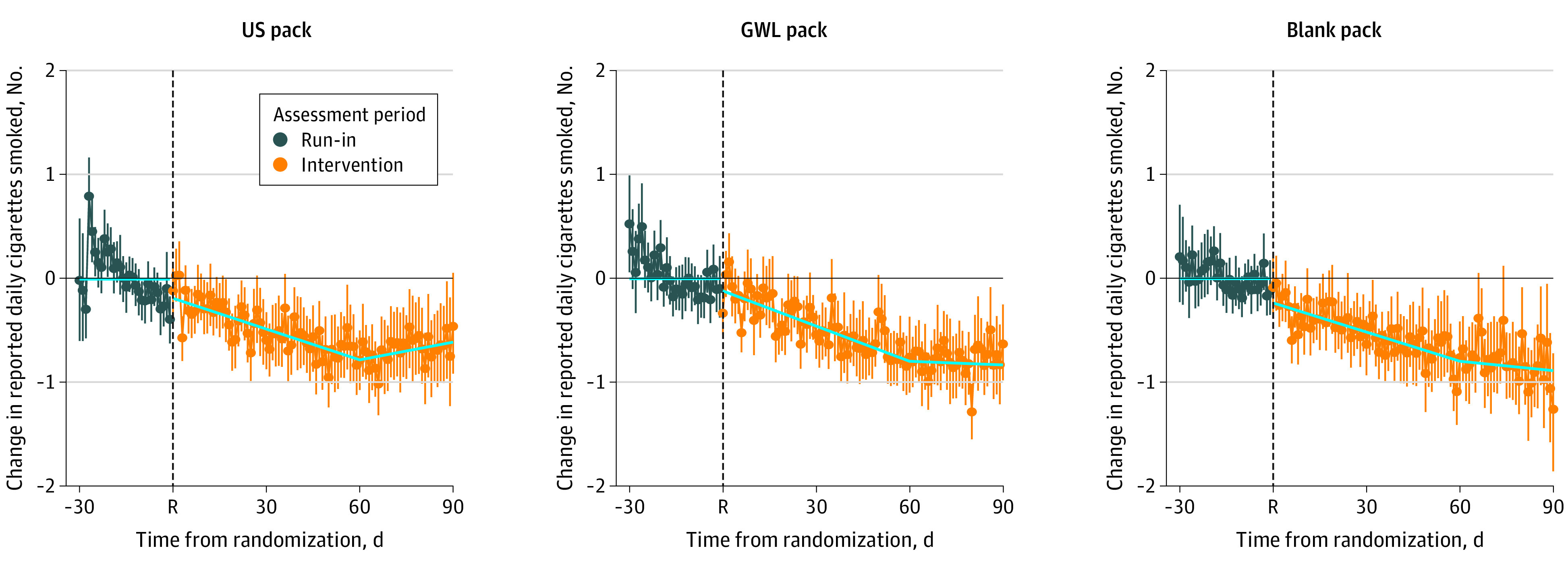
Change in Cigarette Consumption Reported During Daily Ecological Momentary Assessments (EMAs) by Interactive Texting During the run-in period, all groups received the US pack, and, at week 0 (randomization [R]), participants were allocated to a study pack group for the 90-day intervention. Daily cigarette consumption is estimated from the number of cigarettes reported smoked within the last 4 hours captured by the daily interactive text messaging responses. Data were available for 115 US pack participants, 114 graphic warning label (GWL) pack participants, and 124 blank pack participants. Solid line shows mean predictive values from a mixed effects model using compound symmetry covariance structure with run-in slope set to 0 and a knot at 60 days. Vertical segments have observed daily mean changes in number of cigarettes consumed and their 95% CIs. Details are in eTable 6 in Supplement 2.

Daily cigarette consumption reported on the daily EMA texts decreased over the first 2 months of the intervention, with no difference between study groups (Figure 4). Only in the third month was the change in consumption slightly lower in the GWL pack compared with the US pack (eTable 6 in Supplement 2).

### Postintervention Assessments of Smoking Behavior

At the baseline study visit (visit 1), the US pack group reported a mean (SD) of 11.8 (5.6) cigarettes per day compared with 11.2 (6.0) cigarettes per day for the GWL pack group and 12.0 (6.2) cigarettes per day for the blank pack group, with no between-group differences. Saliva cotinine concentrations (in nanograms per milliliter) validated this lack of difference (Table). At the 3-month visit (visit 2), 16 participants (4.6%) were cigarette abstinent for 30 days with no between-group difference. Furthermore, there was no between-group difference in reported cigarette consumption at visit 2, which was approximately 2 cigarettes per day lower than at visit 1 (GWL vs US pack, adjusted mean difference, −0.2 cigarettes per day; 95% CI, −1.8 to 1.3 cigarettes per day). Saliva cotinine concentrations also decreased between study visits, but with no between-group difference (GWL vs US pack, −11.5 ng/mL; 95% CI, −67.8 to 44.8 ng/mL; blank vs US pack, 14.8 ng/mL; 95% CI, −41.6 to 71.1 ng/mL).

**Table.** Prestudy and Poststudy Assessments of Cigarette Consumption and Saliva Cotinine Concentrations

Timing of measure and variable assessed	Study intervention group, mean (SD)			Adjusted difference, mean (95% CI)^a^	
	US pack (n = 109)	GWL pack (n = 114)	Blank pack (n = 118)	GWL vs US pack	Blank vs US pack
Prerandomization visit					
Cigarettes/d, No.	11.80 (5.59)	11.20 (5.99)	12.00 (6.17)	−0.5 (−1.9 to 0.9)	−0.02 (−1.4 to −1.3)
Saliva cotinine level, ng/mL	365.3 (256.5)	337.9 (262.5)	356.3 (250.4)	−22.0 (−92.2 to 48.2)	−13.2 (−82.1 to 55.7)
Change from visit 1 to visit 2^b^					
Quit for 30 d, % (95% CI)	4.7 (2.0 to 10.6)	5.5 (2.5 to 11.5)	3.5 (1.4 to 8.7)	1.1 (0.32 to 3.79)	0.66 (0.17 to 2.56)
Cigarettes/d, No.	−2.0 (3.9)	−2.2 (4.7)	−2.4 (5.5)	−0.2 (−1.8 to 1.3)	−0.4 (−1.9 to 1.1)
Saliva cotinine level, ng/mL	−39.3 (146.2)	−46.9 (169.5)	−23.1 (197.9)	−11.5 (−67.8 to 44.8)	14.8 (−41.6 to 71.1)

Abbreviation: GWL, graphic warning label.

^a^ Adjusted for age, sex, and nicotine dependence.

^b^ Visit 1 was the prerandomization study visit. Visit 2 was the 3-month clinic visit.

## Discussion

In this real-world, randomized clinical trial, we examined the effect of cigarette packs with GWLs on cigarette smokers. We succeeded in encouraging US smokers to purchase cigarettes that were repackaged into either GWL or blank packs and delivered to them, with 96% of those randomized completing their postintervention assessment. Across the first 2 months of the intervention, only those in the GWL pack group reported a consistent decrease in their positive perceptions of the cigarettes that they smoked, although the expected increase in perceived health concerns was not significant. Although there was a significant increase in cognitions about quitting in the GWL group, there was no evidence of increased quitting or reduced consumption, and this was biochemically validated at the postintervention visit.

Tobacco branding on a cigarette pack typically increases a smoker’s perceptions of satisfaction, craving relief, and taste of cigarettes.^6,29^ However, in this study, removal of tobacco branding on the blank pack was not sufficient to decrease these positive perceptions. It was only the GWL pack group that had lower positive perceptions. Such an effect might be expected from psychological experiments that found that the most effective way to reduce affect-based cognitions was to expose individuals to ones with an opposite affect valence.^30^

Health concerns increased in all study groups during the intervention, with only a marginal increase in the GWL pack group. As the theory supporting mandated GWLs expects that their effect will be achieved through increased health concerns, this lack of a marked between-group difference was unexpected. The increase in health concerns among the US pack group is likely associated with the continual interactive text messaging on thoughts about the health consequences of smoking. That the GWL pack did not experience a significant increase these cognitions may reflect the high level of awareness of the health consequences of smoking,^31^ which was also seen in our study. In the real world, daily EMA texts would not occur, which might result in a GWL effect on these health concerns that was not apparent in this study.

Although the GWL group participants were more likely to think about quitting during the study intervention, there was no evidence of increased quitting behavior. Our measure of at least 1 weekly 4-hour smoking abstinence was designed to maximize the possibility of identifying early quitting activity. What was surprising is that the GWL group participants, who were more likely to think about quitting, were not much more likely to report these short smoking abstinences or to report lower daily cigarette consumption. Our study is in line with previous research indicating that intentions to change are rarely sufficient to achieve change in an addictive behavior.^32^

### Limitations

Our repackaging of participants’ cigarettes into GWL packs provided a realistic packaging design; however, home delivery of cigarettes may mean that the results might be different from cigarettes purchased in a store. Before the study, our participants described the GWL packs as aversive.^20^ Given that branded packs were readily available to participants, the study’s 15% per pack discount^19^ was not sufficient to prevent participants from obtaining approximately one-half of their cigarettes from nonstudy sources. Ultimately, the intervention still achieved substantial exposure to the study packs.

The use of EMAs through interactive texting to obtain daily participant self-reports of cognitions and behavior was essential to assess an effect on cognitions close to the time of smoking. However, such continual prompting can itself be an intervention,^33^ as demonstrated by the changes in perceptions of health effects and thoughts about quitting in the US pack group. Although self-reported changes in smoking are a limitation, changes were validated biochemically, which is a major advance on previous studies.

In this study, we chose the Australian standardized packaging for our GWL packs rather than the hybrid packs proposed for implementation in the US. Legal advice recommended using an existing pack design and against manufacturing packs that required us to alter a current tobacco company design.

## Conclusions

In summary, this randomized clinical trial presents strong evidence that GWLs, but not simply the removal of tobacco industry marketing, can counter the appeal of tobacco marketing on cigarette packs and increase cognitions about quitting. However, on their own, they are not a strong enough tobacco control measure to reduce cigarette consumption among those not ready to quit.

## Notes

Supplement 1. Trial Protocol

Supplement 2. **eFigure 1.** Study Design **eFigure 2.** Images of Manufactured Study Packs **eTable 1.** Sample Characteristics by Randomization Group **eTable 2.** Model of Change in Positive Perceptions (Satisfaction, Taste, and Craving) to Pack Cigarettes **eTable 3.** Model of Change in the Perception That Smoking Impacted Your Health and That of Others **eTable 4.** Model of Change in Weekly Quitting Cognitions **eTable 5.** Model of Change in Percent of Participants With at Least One Cigarette Abstinence Period/Week **eTable 6.** Model of Change in Cigarette Consumption Reported During Daily EMAs

Supplement 3. Data Sharing Statement
