# Error in Table

**DOI:** 10.1001/jamanetworkopen.2022.45981

**Published:** 2022-11-18

In the Original Investigation titled “Effect of Graphic Warning Labels on Cigarette Packs on US Smokers’ Cognitions and Smoking Behavior After 3 Months: A Randomized Clinical Trial,”^1^ published August 4, 2021, there were errors in the Table. Footnotes were added to clarify the types of data shown in the 2 right columns. This article has been corrected.^1^
