# Supplementary material for: Effect of Graphic Warning Labels on Cigarette Packs on US Smokers’ Cognitions and Smoking Behavior After 3 Months: A Randomized Clinical Trial
Source: JAMA Netw Open. 2021 Aug 4;4(8):e2121387. doi: 10.1001/jamanetworkopen.2021.21387 (PMC8339936; doi:10.1001/jamanetworkopen.2021.21387)
Supplement: Supplement 1. — Trial Protocol [file jamanetwopen-e2121387-s001.pdf]

UCSD Human Research Protections Program
New Biomedical Application
RESEARCH PLAN

Instructions for completing the Research Plan are available on the [HRPP website.]
The headings on this set of instructions correspond to the headings of the Research Plan.
General Instructions: Enter a response for all topic headings.
Enter "Not Applicable" rather than leaving an item blank if the item does not apply to this project.

Version date: 9/30/2013

1. PROJECT TITLE

Effect of Packaging on Smoking Perceptions and Behavior: A Randomized Trial

2. PRINCIPAL INVESTIGATOR

David Strong, PhD, Associate Professor in the Department of Family and Preventive Medicine
John P. Pierce, PhD, Professor in the Department of Family and Preventive Medicine

3. FACILITIES

Moores Cancer Center
East Campus Office Building

4. ESTIMATED DURATION OF THE STUDY

5 years

5. LAY LANGUAGE SUMMARY OR SYNOPSIS (no more than one paragraph)

We are addressing the impact of cigarette packaging and labeling on consumer perceptions and on smoking behavior; and the effectiveness of graphic warning labels on communicating risk of tobacco products. Smokers handle their packs frequently. Product packaging is a crucial medium by which the tobacco industry communicates product attributes, including reassurances about risk. The industry uses branded imagery on packs to influence consumer perceptions of their product and to reinforce brand loyalty, while at the same time the tobacco control community uses health warning labels to communicate harmful and potentially harmful constituents and risks of tobacco products. The World Health Organization Framework Convention on Tobacco Control (WHO FCTC) is a landmark treaty enacted to ensure that every person is informed of the health consequences, addictive nature and mortal threat posed by tobacco consumption and exposure to tobacco smoke. In 2011, the FDA announced that new larger and more noticeable textual warning statements along with graphic images must appear on cigarette packages and in cigarette advertisements. Five tobacco manufacturers successfully filed suit challenging this FDA regulation. In upholding the district court's finding against the FDA action, Appeals court Judge Janice Jones wrote "FDA failed to present any data -- much less the substantial evidence required under the federal law -- showing that enacting their proposed graphic warnings will accomplish the agency's stated objective of reducing smoking rates." This objective of this project is to undertake rigorous research to provide some of this needed data. The design and extensive objective measurements are significant innovations on the currently published research in this field and should lead to significant advances in tobacco regulatory science.

6. SPECIFIC AIMS

Primary Aim 1: To test whether removing all tobacco industry imagery from the standard US pack (plain packs using current US warning label, white on black) changes consumer perceptions of their cigarettes. We hypothesize that a gap will develop across study groups (AM> PP > ST) with:

a) increased perceptions of risk from tobacco use, decreased perceptions of appeal of packs and decreased subjective reinforcing effects from their tobacco use
b) greater between-group differences during the intervention period than during 12-month follow- up.

Primary Aim 2: To test whether adding large graphic warning labels to the plain packs of Aim 1 (the Australian model pack) increases the perception of harm and potential risk of tobacco products. We hypothesize that over the three months of the intervention, smokers assigned to AM compared to PP or ST will report:

a) increased change in reported awareness and concern about the health hazards of smoking

b) decreased willingness to display the pack in public

Primary Aim 3: To test whether the Australian model packs result in a change in the handling of cigarette packs, consumption patterns and quitting cognitions. We hypothesize that, compared to objective assessment of behavior when handling ST packs, we will observe greater behavioral change with AM packs than PP, in:

a) greater attentional demand to warning label, changes in handling the pack, obscuring of warning labeling during a cigarette offer challenge, greater recall of warning label messaging
b) greater reductions in tobacco use during the 3-month intervention, validated by saliva cotinine.
c) increased reports of motivation and intention to quit during the 12-month follow-up

7. BACKGROUND AND SIGNIFICANCE

The first country to require health warning labels on tobacco packs was the US in 1966. However, there has been little meaningful change in these text-only messages since then and the imagery and branding on many cigarette packs make messages difficult to read. The salience and recall of warning labels improves when the messages are distinguishable from the package design. Larger text-based warnings are associated with increased risk perceptions and improved health knowledge. Following early reports on their effectiveness of graphic warning labels, in 2003, the WHO Framework Convention on Tobacco Control required the use of large, rotating health warnings on all tobacco products, packaging and labeling, recommending that full color pictures or pictograms be mandated. As of the end of 2011, over 39 countries, but not the US, had implemented pictorial health warnings on cigarette packages (FDA, 2012).

Tobacco companies have used cigarette packaging design to downplay the risks of smoking through the use of brand descriptors, pack colors, brand variant names, and numeric tar and nicotine labels (Wakefield et al, 2002). As a result, both adult smokers and youth believe that low tar or light cigarettes are less harmful and less addictive than regular strength brands, and many smokers switch to lower tar brands instead of quitting.

The marketing effectiveness of pack design prompted proposals to introduce plain cigarette packaging. These "standardized" packaging regulations would (1) strip packs of colors, brand imagery, corporate logos, and trademarks; (2) have the brand name in a mandated size, font and location; and (3) feature health warnings and other legally mandated information. Evidence from multiple sources suggests that both plain packs and graphic health warning labels are associated with changes in risk perceptions among smokers and may be associated with increased quitting and a reduction in daily consumption. However, this evidence relies heavily on hypothetical experiments and from population surveys before and after implementation of national policy changes that included changes in package design. Studies where participants have switched to using new cigarette pack designs have been compromised by short exposures, significant participant burden and high attrition. Although there may be significant public health impact from changing pack designs, controlled evaluations of the persistent impact of proposed changes to labeling are unavailable (Hammond et al, 2011). The United States is now one of the few high-income countries that can conduct such trials. In recent decades, the US has been a laggard in using the cigarette pack to inform on the health hazards of smoking. It would appear that the quality of evidence demanded by the Appeals court judge in order to limit the commercial speech of the tobacco industry on cigarette packages in the US, will require rigorous randomized controlled trials among US populations of smokers.

8. PROGRESS REPORT

Not Applicable.

9. RESEARCH DESIGN AND METHODS

9.A. Overview

Much of the current research base on pack design and consumer perceptions has relied upon single-occasion experimental assessment, using hypothetical scenarios to measure expectations, and has relied on images of packs rather than actual packs in cued-reaction tasks. It is now possible to have objective behavioral assessment of real-time consumer interactions with professionally produced cigarette packaging. These objective behaviors can then be connected with mobile technology to allow intensive capture of day-to-day changes in perceptions and tobacco use behavior in the context of the consumers' typical environment. We have licensed from the Australian Government Department of Health, the strongest images of tobacco-related harms currently in use. This unprecedented randomized controlled trial will provide a unique opportunity to examine smokers' acute and persistent adaptations to proposed policy recommendations to change the labels and marketing materials on US cigarette packaging.

The real world experience in Australia demonstrates that smokers can continue to smoke with large graphic warning labels (Wakefield et al., 2008). We will conduct our randomized study by producing packs to specifications used in the real world setting of Australia, and filling them with the cigarette brand of the smoker. This will significantly reduce participant burden and should allow us, with appropriate incentives, to achieve 6 times the exposure to these packs as in the best of previous studies. We will use a conjoint marketing study to predict participant response, including unwillingness to continue with the study exposure. Further, we will use interactive text messaging to measure cognitions in real time rather than rely on recall, thus removing another major bias of currently available studies. Finally, we will use the new Kinect 3-D and Leap motion-sensor technology to capture real-time objective behavioral data of participants display cigarette packs when offering a cigarette to a research associate. All questionnaires will be web-based to minimize participant burden further. Thus, our study should significantly add to the knowledge base for tobacco regulatory science.

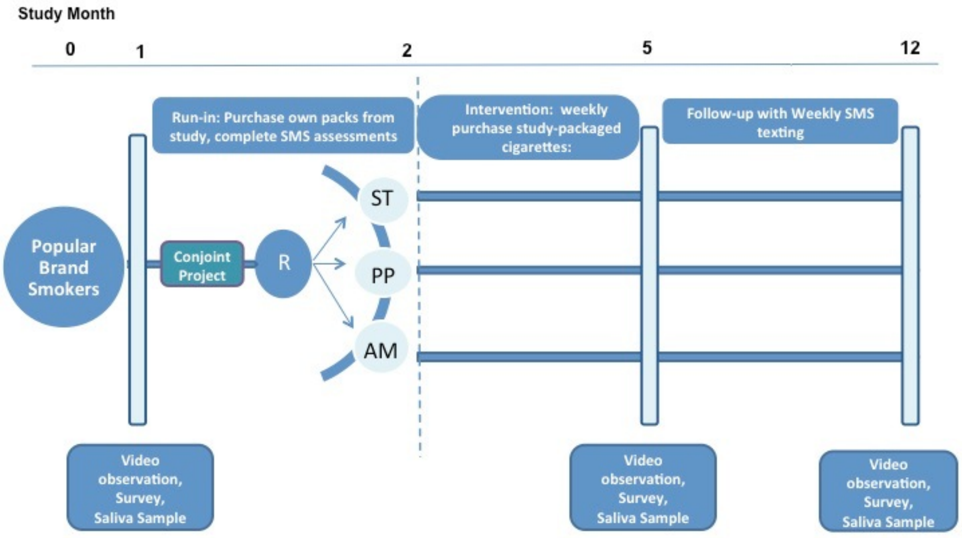

Effect of Packaging on Smoking Perceptions and Behavior: A Randomized Trial

9.B. Approach

9.B.1. Study Overview: Figure 1 shows community recruitment of the study sample of smokers of popular brands of US cigarettes (the top 75% of market share). There will be three major periods within this study.

<u>9.B.1.a Run-In-Period</u> At the initial contact eligible participants will complete all baseline lab and self-report assessments. There will then be a 1-month run-in period during which they will purchase their US branded cigarettes from the study at the study discount price. In order to proceed to randomization and the rest of the study, eligible smokers will need to complete at least 2 purchases from the study as well as the study assessments required for this period.

<u>9.B.1.b. Purchasing Intervention Period</u> After participants have been randomized to purchase their preferred brand of cigarettes from one of 3 cigarette-packaging groups (AM, PP or ST packs), the purchasing intervention will last 3 months, during which smokers will interact with daily text-message and use assigned

packs. We will monitor compliance via text messages, provide multiple points of access to the study vendor and use a price incentive to maximize adherence to purchasing cigarettes from the study.

9.B.1.c. Post-Intervention Follow-up Period, After 3 months, participants will go back to purchasing cigarettes from their usual sources and follow-up will continue through 12 months.

9.B.1.d. Assessment overview: Clinic visits will occur at three time points: at the beginning of the run-in period, at the end of both the purchasing and the post intervention periods. Each clinic visit will be preceded by the completion of a web-based questionnaire, will include collection of a saliva sample and the participant will go through routinized cigarette handling tasks that will be recorded using objective multi-modal sensor-based behavioral monitoring. Lab-based behavioral assessment will allow detailed objective metrics of handling of usual brands, initial behavioral reactions to AM and PP packs, cognitive processing and behavioral interaction with warning labels, and objective assessment of whether smokers change or accommodate handling of packs after 3-months of using ST, AM, or PP. During the run-in period, all participants also will complete a web-based conjoint trade-off study to assess how each element of the AM and PP designs change perceptions of marketing materials, design features, personal relevance of warning messages and value of each product. Throughout each of the study periods, participants will receive a series of interactive text messages as part of our ecological momentary protocol to assess *perceptions* (appeal of packs, subjective effects on product consumption over time), pack use behavior (avoidance, willingness to display), and *effectiveness of warning label communication (*awareness of harms over time, quitting cognitions). Using the self-report assessments at baseline, 5- and 12-month visits, we will assess the following primary domains: a) participant characteristics, including potential moderators; b) marketing materials: salience, receptivity, and perceived attributes; c) tobacco use effects and cognitions; d) environmental influences. This study will provide objective behavioral data, detailed assessment of the impact of packs in the context of smokers' typical environment, and allow these acute and long-term effects to be evaluated in a randomized controlled trial.

9.B.2. Recruitment: We will recruit 450 current smokers who use on of the five most common brands as their usual brand and who live in the San Diego area. Standard media announcements will be used to recruit for this study including print, online bulletin boards, social media, website and community outreach strategies will be used to recruit. We also have ongoing recruitment opportunities with email and print notifications within UCSD Family Medicine clinics serving approximately 30,000 patients through more than 150,000 office visits. Dr. Nodora will liaison with community networks of providers throughout the region to extend recruitment efforts to community health agencies.

9.B.3. The Initial Clinic Visit: Participants will attend a baseline assessment meeting at the UCSD CTRI. During this clinic visit, staff will provide them with study details and allow them to handle the packs for the different study groups. Participants will provide a saliva sample for later cotinine analysis and complete a short survey of their usual cigarette purchasing behavior (including frequency of purchase), their current smoking pattern, the last time they smoked a cigarette other than their usual brand, quitting history and readiness to quit as well as the questions from the Australian multiple cross-sectional survey. They will be introduced to the study vendor website for purchase of discounted cigarettes and commit to using this website as their only vendor for cigarettes for the next 4 months (1-month run-in and 3-month intervention).

9.B.4. Behavioral Assessment of Participant Interactions With Packs: We will employ the extensive 3D multimodal data collection capabilities offered by the Microsoft Kinect sensor (http://www.kinect4windows.org) and the Leap motion device (http://www.leapmotion.com) to augment video analysis during a first exposure to each pack design as well as detail changes in routinized behavior in response to intervention, and persistence of effects over time. The combination of the sensor tracking capabilities will allow us to quantify behaviors (body, finger and hand positions), visual attention (head direction), facial expressions (valence), verbal communication (directional audio) and pack movements (e.g. % of time oriented to side with warning label; % of time obscured). These indicators can be paired with participant reports to assess impact on positive (approach) and negative (avoidance) reinforcing qualities of each pack and warning label design. Metrics obtained will be used to predict real-time changes in perceptions, report of tobacco-related harms, compliance with study pack use, and tobacco use behaviors during the three-month study evaluation period.

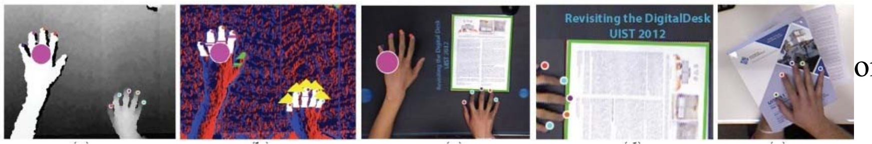

9.B.5. The Conjoint Trade-off Project: Participants will then complete a web-based conjoint analysis designed by consultant, Dr Dimofte who is experienced in the conduct of these studies. Participants will complete a series of web-based choices between digitally altered images of packs at different price points, in a fractional factorial design. Each choice will ask the participant to rank a small subset of the possible packs and to provide individual ratings of pack images for personal relevance, believability, and aversiveness. The study will estimate the relative importance placed on each attribute, which will be of independent interest to the scientific community.

9.B.6. Study Run-in Period: We will use a 1-month run-in period prior to study enrollment. During this period, participants will be required to complete the TQ and text messaging assessment. Further, they will need to make at least 2 purchases of their usual brand of US packaged cigarettes from our study vendor (at a 10% discount over their usual price-assessed earlier). We will use text messaging on a pre-agreed schedule to prompt each purchase with a link to secure study hosted web-order forms. Successful completion of this run-in phase will be required in order for a participant to be considered for randomization. We will use the number of packs purchased/week as a flag for the maximum packs that can be purchased during the intervention to eliminate the possibility of participants reselling cigarettes for profit. Any higher amount requested will trigger a study investigatory contact and approval.

9.B.7. Cigarette Products: We will purchase standard brand cigarettes from local vendors and coordinate repackaging with our Consultant team. We will fill PP and AM packages with US branded cigarettes. We have worked previously with the company that does the re-packaging and has evidence that smokers do not differentiate the final product from the usual industry product.

9.B.8. Randomization to the study arms: We will use a permuted blocks randomization scheme to allocate participants to one of 3 study arms. We will stratify on 2 categories of total consumption history (+/- >5 pack years smoked total) crossed with 2 categories of quitting history (+/- 7+ days abstinence in past year).

9.B.9. Assessment through SMS Text Messaging: We have partnered with HealthCrowd, a vendor experienced in clinical research to provide 2-way SMS messaging with participants. This validated method will allow us to track and report the performance of participants, and allow participants to alert study staff to needs for product delivery, without reliance upon scheduled phone contacts. We will use SMS to flexibly administer both weekly or daily behavioral assessments.

9.B.10. Using Pricing as a Motivational Tool: The study will offer all cigarettes at a discount from the usual price at local discount stores, as an incentive to use study cigarettes. For participants that have not fulfilled their expected purchase amount within a week of their scheduled purchase, the study will contact them and

investigate whether they have quit or significantly reduced their smoking. Should the reason be that they purchased from another supplier, we will use a further price incentive (additional 10% discount) to encourage them to stay in the study intervention; participants will not be aware of this option in advance. If required, we are prepared to discount the cigarettes as much as 50% to maintain participants on the study protocol. However, we will not provide these participants with MORE packs per week than they smoked during the one month run-in period to reduce the potential risk of entrepreneurs re-selling cigarettes for profit.

9.B.11. Study Questionnaire: We will assess tobacco use behavior, cognitions, and affect using nationally validated measures drawn from the FDA/NIDA sponsored PATH survey (Drs Strong, Pierce and Messer are members of the Scientific Investigators group). This will assure compatibility with other large federally sponsored surveys as PATH measures have been harmonized with the Tobacco Use Supplement to the Current Population Survey and the National Health Interview Survey as well as questions from the Australian surveys conducted by consultant Dr Wakefield. We will use the modular web-based survey (TQ).

9.B.12 Saliva Sample: *Salivary cotinine* will be used to measure exposure to nicotine, which may stem from changes in either cigarette or NTP consumption. Saliva will be collected via passive drool into 2mL cryovials, and a minimum 75 ul saliva collection volume immediately verified. Salivary cotinine will be biochemically analyzed in duplicate using Enzyme-Linked Immuno Sorbent Assay (ELISA) kit from Salimetrics, USA.

9.B.13 General statistical analysis plan. The study team has extensive experience presenting analyses from longitudinal data and includes strong biostatistical expertise (**Messer** and **Strong**). We are confident that we will use rigorous and up-to-date inferential methods, while retaining interpretability of the analysis and results. For each aim and hypothesis, we will pre-specify the outcome measure and time point, and the predictors of interest. Models will include the stratification variables for the randomization (consumption level and quitting history). Models will control for a standard set of potential confounders, including age, gender, race/ethnicity, peer and family influences, addiction level, and presence of anxiety disorders. Primary analyses will use logistic regression, a linear, or generalized linear model depending on whether the outcome of interest is binary, continuous or ordinal-scaled. When using data across time points, if needed, we will use a random effects model or generalized estimating equations to control for within subject correlation, in a repeated measures framework. **Power and sample size** With 450 subjects we will have 150 in each arm, Standard US pack (ST), Plain pack (PP), and Plain pack+Graphic warning (Australian Model: AM). We assume 20% dropout, occurring uniformly over the study period. For each outcome measure, the primary effects of interest are the contrasts ST-(PP and AM) and PP–AM, with the expectation that the AM effect will be larger than the PP. We conducted power analysis for the contrast with the smallest sample sizes (PP-AM), using a Fisher exact test or a 2 sample t-test for comparison of effects at the end of study; and, for comparison of in within-subject trends over time, by simulation using a generalized linear mixed effects models (GLMM). We aimed to estimate the effect size we can detect with 80% power, using a 2-sided test of hypothesis at significance level 0.025 to correct for the 2 primary contrasts of interest. For a longitudinal change measured over the three-month intervention period, results from 1000 simulations suggest power >0.85 for expected medium effect sizes in mean differences.

9.B.14 Timeline

We anticipate publishing a series of papers in the academic literature, and the project team will hold biweekly meetings (inviting wide participation) to discuss progress and seek feedback. The project PIs will prepare quarterly progress reports. Study results will be presented in national and international scientific meetings.

10. HUMAN SUBJECTS

Participants enrolled in this study will be drawn from daily smokers in San Diego County. The study will randomize 450 community dwelling daily smokers, otherwise healthy, aged from 21-50 years. The study population will not include special classes of patients (e.g., fetuses, neonates, pregnant women, children,

prisoners, and institutionalized individuals). The study will have a run-in period of one month and participants will need to demonstrate that they can comply with study assessment procedures before they will be eligible to be randomized into the study groups.

Inclusion Criteria:

- 21-50 years of age
- A current resident of San Diego County
- Daily smokers of at least 5 cigarettes/day who are classified as in the precontemplator stage of change (i.e. do not intend to quit smoking in the next 6-months)
- Regular smokers of the five most popular US cigarette brands
- Have a cell phone with unlimited text messaging service

Exclusion Criteria:

- Non-Daily cigarette smokers and daily smokers of alternative brand cigarettes
- Marked organic impairment or unstable medical problems (such as a seizure disorder)
- Current pregnancy or intent to become pregnant during the next 12 weeks.

11. RECRUITMENT AND PROCEDURES PREPARATORY TO RESEARCH

Study personnel will recruit 450 current smokers who use one of the five most popular brands of cigarettes as their usual brand and who live in the San Diego area. Standard media announcements for this study including print, online bulletin boards, social media, website and community outreach strategies will be used to recruit. We also have ongoing collaborations through research funded by the California Tobacco Related Disease Research Program (21XT-0076, PI: Strong) and recruitment access within UCSD Family Medicine clinics serving approximately 30,000 patients through more than 150,000 office visits. At the initial contact, assessors will describe the new study and will obtain verbal consent to enroll. Those who consent to enroll will be screened, invited to attend a baseline study visit, and directed to the web-based baseline survey after verification of electronic contact information. Participants will be given a unique ID and password to access the online survey. Informed consent to complete each survey will be imbedded into the online survey form. Written informed consent will be obtained at the initial study visit with consent procedures approved by UCSD IRB. Additional contacts (to remind participants to complete surveys) will be made via different modes including email, phone and text messaging.

12. INFORMED CONSENT

Participants will provide informed consent in person. Informed consent will be obtained in writing, and the research assistant will explain the consent procedures. The informed consent document will explain in simple terms, before the participant is entered into the study, the risks and benefits to the participant. The informed consent document will contain a statement that the consent is freely given, that the participant is aware of the risks and benefits of entering the study, and that the participant is free to withdraw from the study at any time. Consent will be obtained after a thorough explanation of the study by research staff and an opportunity for the participant to ask questions about the study. The IRB-approved consent form will be signed and dated by the participant and investigator. The signed informed consent document will be retained with study records. Each participant will be given a copy of his or her signed informed consent. He/she will also be given a letter from the investigators with such information as to how to schedule appointments and how to reach the investigators.

13. ALTERNATIVES TO STUDY PARTICIPATION

The alternative to study participation is to not participate.

14. POTENTIAL RISKS
The study will provide an incentive for the smoker to purchase their cigarettes from the study team rather than from a vendor in the community. This price reduction may make it less likely that the individual will quit smoking during the three months of the active intervention. Based on the experiences of the investigative team in research with smokers not intending to quit, we estimate that smokers who report that they do not intend to quit in the next 6 months have 0.03 chance of making a successful change during this time period. The price decrease is estimated to reduce this probability of 0.027 over a limited 3 month period. We expect that this will be outweighed by the effects of the change in package design for two of the study groups, as both the plain packs group and the plain packs with large warning labels groups are expected to be much more influenced to quit by the package design features of the study. We will provide ongoing monitoring of plans to quit as described below. Should a participant wish to quit smoking at any point during the study they will be provided with options of receiving print or electronic materials describing strategies to quit smoking ('Clearing the Air') and sources of smoking cessation services including no-cost services available through the California Smokers Helpline.<br><br>During the clinic visit, the participant will be observed with Kinect and Leap motion video camera sensors and recordings will be captured throughout the behavioral assessment portion of the interview. Prior to the interview, the participant will be informed that this is done to capture their interactions with the marketing materials. The text of the informed consent will include a clear explanation that any images obtained from the video will not be used outside of the study setting unless there is additional informed consent.<br><br>There is little likelihood of any physical, social or legal risk as a result of participation in this research project. There is a slight possibility that participants might be psychologically stressed from completing the web-based surveys. The consent language imbedded in the web-based survey will remind participants that they may skip over any question that they are uncomfortable answering, and that they may stop the survey at any time and/or withdraw from the study at any time without penalty.<br><br>There are potential risks to confidentiality if the security of the relational databases should be compromised. These databases will be password protected as described above and reside on an access-controlled virtual local area network kept in a secure site within the Moores UCSD Cancer Center. The potential risks to participants from this research are thought to be minimal.

15. RISK MANAGEMENT PROCEDURES AND ADEQUACY OF RESOURCES
Prior to each weekly purchase of cigarettes from the study team, we will ask the smoker's readiness to quit. Participants also will be given instructions to use text messaging tools to contact study staff at any time for information about quitting or referral to smoking cessation services. Information about cessation services is available via print material, website links, phone contact information via text message, and access to an on-call clinical psychologist on the study staff. Should a person express any desire to quit, this will be recorded as a potential endpoint (possible successful quit) and the patient will be provided with a list of a broad range of available cessation services in the San Diego community, provided with access to NCI's Clearing the Air booklet and directed to contact the no-cost California Smokers Helpline for assistance in quitting. Data containing participant identifiers (if any) will be securely stored and protected from theft and/or exposure by the use of passwords and encryption (when data are stored electronically) and locked cabinets (when the data are stored on any other transportable medium). Maximum effort will be applied to store as little sensitive information as possible on any portable hardware. All study materials will be kept in locked files in locked file rooms with exclusive access by study staff only. All results and correspondence produced from this study will only describe patient results in aggregate. Any individual patient material in a physical medium will be shredded at the end of the study. Patient level data will not be disclosed, copied, or transmitted in total or in part to anyone not connected with the approved protocol.

It is considered highly unlikely that there will be any adverse health events during this study. In the event of an error or adverse event during the study period, we will notify the Data Safety and Monitoring Committee of the Moores UCSD Cancer Center and follow their recommendations who will have the responsibility to rectify the situation or assess harm.

16. PRIVACY AND CONFIDENTIALITY CONSIDERATIONS INCLUDING DATA ACCESS AND MANAGEMENT

Data collection methods will include data obtained specifically for research purposes, including: web-based surveys and study assessment questionnaires; electronic text message questionnaires; digital images captured during video assessment interviews and saliva specimens for cotinine analysis. In addition, participants will utilize a study specific vendor website for purchasing of cigarette study product and will be prompted to purchase using text messaging on mobile phones. We will also make use of existing records and data, including: tobacco education materials and materials from publications and presentations.

Information collected from participants will be coded in a manner that precludes their identification. Data collected using survey, text message, and digital image files will be stored in locked file cabinets; with documentation containing identifying information kept separately from data that is coded by identification numbers. The roster linking participants with their identification number will be stored in a file drawer separate from the survey, text message, and image files. All data collected and entered into the computer will be identified only by a coded identification number and email accounts generated by study personnel.

Data that is collected via web and text messaging will be subject to additional security measures. Physical access to the computers will be restricted and conform to the security standards of UCSD and Moores Cancer Center. Participant data will be encrypted and will utilize a granular permissions model incorporating context-based, role-based and user-based access and authentication controls, as specified by the HIPAA Privacy and Security Rules, as well as industry-standard best practices. System Logs will track various types of system access including, but not limited to, “log in,” “communication,” “data view,” and “data modification” events. Electronic Protected Health Information (ePHI) will be isolated (e.g. 18 types of identifiers) and de-identified from coded research data such that only users with sufficient privileges (e.g. project staff in the participant contact role) will have access to confidential, personal information. All confidential, personal patient-reported information will be housed on Moores Cancer Center secured machines. All data will be backed up daily.

17. POTENTIAL BENEFITS

Participants will be contributing to scientific knowledge about the impact of cigarette packaging interventions on risk perception and associated cigarette use and quitting behaviors. This research will aid national governments, states, and communities in determining which cigarette health warning and packaging interventions are most effective in reducing tobacco use and increasing quitting behavior while also providing them with evidence-based regulatory science in support of policy decisions for tobacco control and use reduction. Study participants may benefit significantly from what we learn regarding influences on smoking behavior. In view of the substantial potential benefits and the minimal risk involved, the risk/benefit ratio is highly favorable.

18. RISK/BENEFIT RATIO

The risks to participants are judged to be acceptable relative to the anticipated benefits. By participating in this project, participants may benefit from the comprehensive behavioral assessment of their smoking patterns. If participants elect to quit smoking, withdrawal symptoms during smoking cessation may be unpleasant, but the health benefits to participants of successfully remaining abstinent are substantial. Consequently, the risk to benefit ratio in the proposed study appears to be acceptable.

19. EXPENSE TO PARTICIPANT

There will be no expense for participation except for providing transportation to the study site.

20. COMPENSATION FOR PARTICIPATION

Participants will be compensated $20 if they successfully complete the initial baseline visit and provide a saliva sample during the run-in period. During run-in they will purchase their usual brand of cigarettes at a 10% discount from the study vendor. Following randomization, they will continue to purchase discounted cigarettes but with packaging according to study design for the intervention period; however, should they miss a scheduled purchasing point and indicate that they have purchased their usual brand elsewhere, they will be offered an additional discount to incentivize them to get back on the study protocol. After completion of the intervention period, smokers will no longer have access to discounted cigarettes from the study vendor. At 5 and 12 months, participants will receive $40 for completion an additional web-based survey and provision of a saliva sample at each of these assessments. Finally, if they have completed all assessments in the study, they will receive an additional $60 at the end of the study. Thus, the total incentive for all participants in the study, regardless of study arm, will be $160.

21. PRIVILEGES/CERTIFICATIONS/LICENSES AND RESEARCH TEAM RESPONSIBILITIES

David Strong, PhD, Lead PI: Dr. Strong has more than 15 years of experience in tobacco-related clinical and public health research. Dr. Strong will serve as Lead PI and provide primary oversight of all aspects of the study. He will share responsibility for the overall project with Dr. John Pierce. Additionally, Dr. Strong will participate in analyses of study data, presentation of study results, and developing manuscripts for submission to peer-review journals.

John Pierce, PhD, Co-PI: Dr. Pierce is the Director for Population Sciences at Moores UCSD Cancer Center and is a Distinguished Professor in the Department of Family and Preventive Medicine at UCSD. He is broadly trained in epidemiology, psychology and communications research and has been working with large scale data sets on tobacco attitudes knowledge and behavior for 20 years. Dr. Pierce will share responsibility of the overall project with Dr. David Strong. Additionally, Dr. Pierce will provide his expertise and will participate in interpretation of study data and the preparation of study findings and papers.

Karen Messer, PhD, Co-Investigator: Dr. Messer is a Professor in the Department of Family and Preventive Medicine, Division of Biostatistics, at UCSD and is director of the UCSD Moores Cancer Center Biostatistics Shared Resource, a group of 13 faculty and staff dedicated to cancer research housed in dedicated office space at the Cancer Center. She will be responsible for methodological and statistical oversight of the study design and will provide active input at all stages of the study through interaction at regular study meetings. Dr. Messer will assist in manuscript preparation and presentation of study findings at national meetings

Jesse Nodora, PhD, Co-Investigator: will work closely with the research team to address participant recruitment and retention as well as cultural factors related to Hispanic/Latino participants. He has more than 25 years of experience working with Southwest Latino populations and 10 years' experience in tobacco control practice. The proposed research fully uses his health promotion experience, bilingual (English/Spanish) and bicultural (Mexican/ American) background, and multi-national socio-behavioral research skills.

Nadir Weibel, PhD, Co-Investigator: Dr. Weibel is Reseach Scientist in Department of Computer Science and Engineering at UC San Diego, and a Research Health Science Specialist at the VA San Diego Health System. Dr. Weibel will develop and oversee the real-time lab-based video and detailed sensor-based assessments of participant behavioral interactions with the cigarette packs. He will conduct analysis of session data and compile databases for predictive analytics using the longitudinal assessments. He will generate manuscripts and

assist in data analysis and presentation of study results.

TBN, Post-Doctoral Employee: They will assist in all aspects of the study including recruitment, enrollment, assessment, and follow-up of participants. They will assist with data preparation and data analysis throughout the study. They will be actively involved in manuscript preparation alongside study investigators.

Susan Faerber, BA, Project Manager: Ms. Faerber will direct the recruitment and retention incentive program to maximize retention. Ms. Faerber designed and implemented the successful incentive program for the WHEL Study (a long-term study with dietary intervention). She will participate in regular study meetings and interact with study management software systems to maintain detailed databases and reports of participants flow through the study.

Susan Wancewicz, BA, Data Manager: Ms. Wancewicz will perform all aspects of data management, under the direction of Dr. Strong, including, data organization, storage, backup, and quality control, as well as conducting data cleaning routines, and generating data code books.

Nicole Campbell, BA, Project Coordinator: Ms. Campbell will be responsible for coordinating day-to-day operations of the study. Responsibilities will include managing participant recruitment strategies, enrollment, and schedule of timed participant contacts. She will attend weekly study meetings and assist in the preparation of study-related presentations and facilitate document management of study activities.

Eliza Ferguson, BS, Project Coordinator: Ms. Ferguson will be responsible for coordinating assessments including interacting with HealthCrowd to develop and deploy the intensive SMS daily assessments. She will work as a liaison with vendors producing study materials and ensure coordination of distribution of pack supplies. She will attend weekly meetings, monitor and report vendor-related study activities, and assist in manuscript editing and document preparation for publications.

22. BIBLIOGRAPHY

FDA. Overview: Cigarette Health Warnings. 2012 [cited 2012 November]; Available from: http://www.fda.gov/TobaccoProducts/Labeling/ucm259214.htm.

Borland, R., et al., *Impact of graphic and text warnings on cigarette packs: findings from four countries over five years.* Tobacco Control, 2009. **18**(5): p. 358-364.

Brown, K.G., et al., *Graphic imagery is not sufficient for increased attention to cigarette warnings: The role of text captions.* Addiction, 2012.

Hammond, D., *Health warning messages on tobacco products: a review.* Tobacco Control, 2011. **20**(5): p. 327-337.

Wakefield, M., et al., *The cigarette pack as image: new evidence from tobacco industry documents.* Tobacco Control, 2002. **11**: p. I73-I80

Wakefield, M.A., et al., *Impact of tobacco control policies and mass media campaigns on monthly adult smoking prevalence.* American Journal of Public Health, 2008. **98**(8): p. 1443-1450.

23. FUNDING SUPPORT FOR THIS STUDY

This study is pending funding from the NIH from the following mechanism: RFA-OD-13-011.

24. BIOLOGICAL MATERIALS TRANSFER AGREEMENT

Not applicable.

25. INVESTIGATIONAL DRUG FACT SHEET AND IND/IDE HOLDER

Not Applicable.

26. IMPACT ON STAFF
None.
**27. CONFLICT OF INTEREST**
None.
**28. SUPPLEMENTAL INSTRUCTIONS FOR CANCER-RELATED STUDIES**
Not applicable.
**29. OTHER APPROVALS/REGULATED MATERIALS**
Not applicable.
**30. PROCEDURES FOR SURROGATE CONSENT AND/OR DECISIONAL CAPACITY ASSESSMENT**
Not applicable.

Version date: May 11, 2011
