# Supplementary material for: Effect of Graphic Warning Labels on Cigarette Packs on US Smokers’ Cognitions and Smoking Behavior After 3 Months: A Randomized Clinical Trial
Source: JAMA Netw Open. 2021 Aug 4;4(8):e2121387. doi: 10.1001/jamanetworkopen.2021.21387 (PMC8339936; doi:10.1001/jamanetworkopen.2021.21387)
Supplement: Supplement 2. — eFigure 1. Study Design eFigure 2. Images of Manufactured Study Packs eTable 1. Sample Characteristics by Randomization Group eTable 2. Model of Change in Positive Perceptions (Satisfaction, Taste, and Craving) to Pack Cigarettes eTable 3. Model of Change in the Perception That Smoking Impacted Your Health and That of Others eTable 4. Model of Change in Weekly Quitting Cognitions eTable 5. Model of Change in Percent of Participants With at Least One Cigarette Abstinence Period/Week eTable 6. Model of Change in Cigarette Consumption Reported During Daily EMAs [file jamanetwopen-e2121387-s002.pdf]

# Supplemental Online Content

Strong DR, Pierce JP, Pulvers K, et al. Effect of graphic warning labels on cigarette packs on US smokers' cognitions and smoking behavior after 3 months: a randomized clinical trial. *JAMA Netw Open*. 2021;4(8):e2121387. doi:10.1001/jamanetworkopen.2021.21387

**eFigure 1.** Study Design

**eFigure 2.** Images of Manufactured Study Packs

**eTable 1.** Sample Characteristics by Randomization Group

**eTable 2.** Model of Change in Positive Perceptions (Satisfaction, Taste, and Craving) to Pack Cigarettes

**eTable 3.** Model of Change in the Perception That Smoking Impacted Your Health and That of Others

**eTable 4.** Model of Change in Weekly Quitting Cognitions

**eTable 5.** Model of Change in Percent of Participants With at Least One Cigarette Abstinence Period/Week

**eTable 6.** Model of Change in Cigarette Consumption Reported During Daily EMAs

This supplemental material has been provided by the authors to give readers additional information about their work.

© 2021 Strong DR et al. *JAMA Network Open.*

## eFigure 1. Study Design

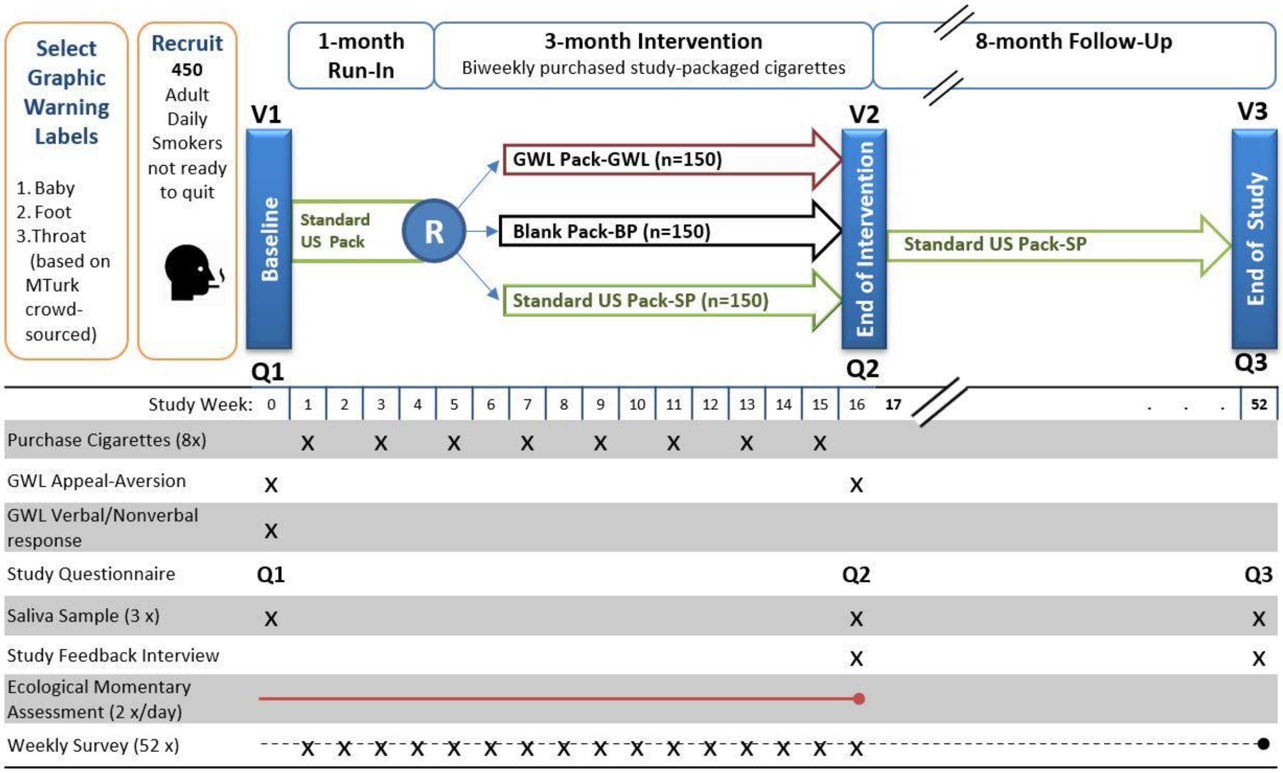


Reprinted from Pierce JP, Strong DR, Stone MD, et al. Real-world exposure to graphic warning labels on cigarette packages in US smokers: The CASA randomized trial protocol. *Contemp Clin Trials*. 2020;98:106152.

© 2021 Strong DR et al. *JAMA Network Open.*

## eFigure 2. Images of Manufactured Study Packs

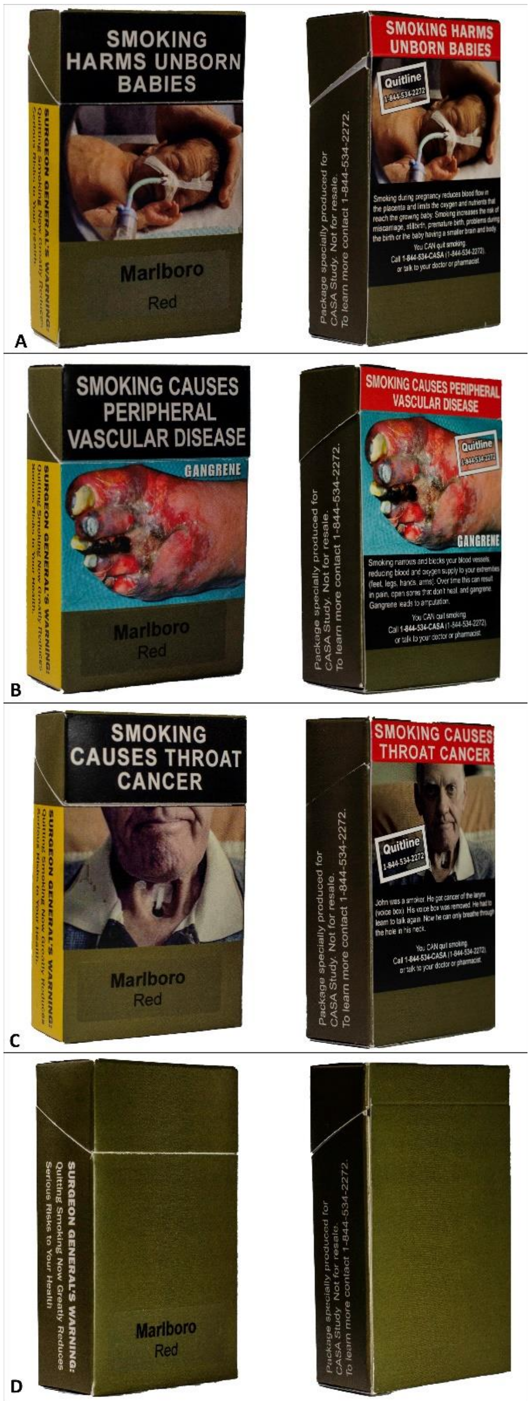


Legend: Images of Manufactured Study Packs: (A) Neonatal Baby[a], (B) Foot Gangrene [a], (C) Throat Cancer [a], (D) Blank Pack.
Reprinted from Pierce JP, Strong DR, Stone MD, et al. Real-world exposure to graphic warning labels on cigarette packages in US smokers: The CASA randomized trial protocol. *Contemp Clin Trials*. 2020;98:106152.
[a] © Commonwealth of Australia

| eTable 1. Sample Characteristics by Randomization Group | | | | | |
|---|---|---|---|---|---|
| **Characteristic** | Overall (*n*=359) | Standard US (*n*=116) | GWL Pack (*n*=118) | Blank Pack (*n*=125) | *P*-Value |
| **Age at study entry, y, Mean (SD)** | 39.47 (11.88) | 39.32 (11.63) | 39.26 (12.19) | 39.62 (11.90) | .97[a] |
| **Gender Female, *n (%)*** | 195 (54.3%) | 71 (61.2%) | 55 (46.6%) | 69 (55.2%) | .079[b] |
| **Race/Ethnicity NH White, *n (%)*** | 245 (68.2%) | 81 (69.8%) | 78 (66.1%) | 86 (68.8%) | .82[b] |
| **Education College degree, *n (%)*** | 149 (41.5%) | 53 (45.7%) | 48 (40.7%) | 48 (38.4%) | .51[b] |
| **Income* <$50,000/year, *n (%)*** | 143 (39.8%) | 49 (42.2%) | 46 (39.0%) | 48 (38.4%) | .23[b] |
| **Nicotine Dependence, *Mean (SD)*** | 3.84 (2.28) | 3.86 (2.26) | 3.69 (2.29) | 3.90 (2.30) | .77[a] |
| **Last 7 days Cigarettes/day, *Mean (SD)*** | 12.08 (9.25) | 13.04 (10.19) | 11.83 (8.69) | 12.86 (8.89) | .55[a] |

*Income question was not asked of first 99 people in study

*Note.* [a] Calculated using the ANOVA test. [b]Calculated using the $\chi^2$ test.

Reprinted from Pierce JP, Strong DR, Stone MD, et al. Real-world exposure to graphic warning labels on cigarette packages in US smokers: The CASA randomized trial protocol. *Contemp Clin Trials*. 2020;98:106152.

© 2021 Strong DR et al. *JAMA Network Open.*

## eTable 2: Model of Change in Positive Perceptions (Satisfaction, Taste, and Craving) to Pack Cigarettes

| | Estimate | Std. Error | | t-value | p-value |
|---|---|---|---|---|---|
| **Age** | | | | | |
| **Age <45** | -- | -- | | -- | -- |
| **Age 45+** | -0.002 | 0.019 | | -0.104 | 0.917 |
| **Gender** | | | | | |
| **Female** | -- | -- | | -- | -- |
| **Male** | -0.004 | 0.018 | | -0.218 | 0.828 |
| **Nicotine Dependence** | 0.003 | 0.004 | | 0.856 | 0.392 |
| **Positive Perceptions Pre-Randomization** | | | | | |
| **Pre-Randomization Level (US Pack)** | -0.006 | 0.027 | | -0.243 | 0.808 |
| **Pre-Randomization Level (GWL Pack)** | -0.003 | 0.027 | | -0.122 | 0.903 |
| **Pre-Randomization Level (Blank Pack)** | -0.011 | 0.026 | | -0.406 | 0.685 |
| **Positive Reactions During Intervention** | | | | | |
| **Post-Randomization (US Pack)** | 0.002 | 0.027 | | 0.082 | 0.935 |
| **Post-Randomization (GWL Pack)** | -0.011 | 0.027 | | -0.420 | 0.675 |
| **Post-Randomization (Blank Pack)** | -0.005 | 0.027 | | -0.191 | 0.848 |
| **Change Rate in Positive Perceptions (US Pack, reference)** | | | | | |
| **During first 2 months** | -0.001 | <0.0001 | | -4.510 | <0.0001 |
| **Additional slope from 3rd month** | 0.002 | 0.001 | | 3.020 | 0.003 |
| **Differences in Change Rate in Positive Perceptions (Pack x Month interaction)** | | | | | |
| **US Pack (During first 2 months)** | -- | -- | | -- | -- |
| **GWL Pack (During first 2 months)** | -0.002 | <0.0001 | | -5.710 | <0.0001 |
| **Blank Pack (In first 2 months)** | 0.000 | <0.0001 | | 1.230 | 0.218 |
| **US Pack (Additional slope from 3rd month)** | -- | -- | | -- | -- |
| **GWL Pack (Additional slope from 3rd month)** | 0.001 | 0.001 | | 0.526 | 0.599 |
| **Blank Pack (Additional slope from 3rd month)** | 0.001 | 0.001 | | 0.689 | 0.491 |

Note: Estimate = regression model estimate; Std. Error = Standard Error of model estimate; Models estimate relationships with positive perceptions scores formed from 3 questions rated daily on a 4-point scale and normalized to reflect differences from an average rating during the 1 month baseline period. Rates of change reflect increments in normalized scores each day during the 12-week intervention period. Mean differences between GWL vs US averages during treatment were divided by the pooled standard deviation for a standardized effect of –0.07 (95% CI, -0.03, -0.12).

© 2021 Strong DR et al. *JAMA Network Open.*

## eTable 3: Model of Change in the Perception That Smoking Impacted Your Health and That of Others

| | Estimate | Std. Error | | t-value | p-value |
|---|---|---|---|---|---|
| **Age** | | | | | |
| **Age <45** | -- | -- | | -- | -- |
| **Age 45+** | 0.08 | 0.04 | | 2.03 | 0.04 |
| **Gender** | | | | | |
| **Female** | -- | -- | | -- | -- |
| **Male** | -0.09 | 0.04 | | -2.47 | 0.01 |
| **Nicotine Dependence** | 0.01 | 0.01 | | 1.38 | 0.17 |
| **Health Perceptions Pre-Randomization** | | | | | |
| **Pre-Randomization Level (US Pack)** | -0.06 | 0.06 | | -1.07 | 0.29 |
| **Pre-Randomization Level (GWL Pack)** | -0.06 | 0.06 | | -1.11 | 0.27 |
| **Pre-Randomization Level (Blank Pack)** | -0.05 | 0.06 | | -0.87 | 0.38 |
| **Health Perceptions During Intervention** | | | | | |
| **Post-Randomization (US Pack)** | -0.03 | 0.06 | | -0.54 | 0.59 |
| **Post-Randomization (GWL Pack)** | 0.06 | 0.06 | | 0.97 | 0.33 |
| **Post-Randomization (Blank Pack)** | -0.04 | 0.06 | | -0.65 | 0.52 |
| **Change Rate in Health Perceptions (US Pack, reference)** | | | | | |
| **Weeks during intervention** | 0.01 | <0.001 | | 4.07 | <0.001 |
| **Differences in Change Rate in Health Perceptions (Pack x Week interaction)** | | | | | |
| **US Pack (Weeks during intervention)** | -- | -- | | -- | -- |
| **GWL Pack (Weeks during intervention)** | 0.01 | <0.001 | | 1.83 | 0.07 |
| **Blank Pack (Weeks during intervention)** | 0.00 | <0.001 | | -0.42 | 0.67 |

Note: Estimate = regression model estimate; Std. Error = Standard Error of model estimate; Models estimate relationships with health perceptions formed from 2 questions rated weekly on a 4-point scale and normalized to reflect differences from an average rating during the 1-month baseline period. Rates of change reflect increments in normalized scores each week during the 12-week intervention period. Mean differences between GWL vs US averages during treatment were divided by the pooled standard deviation for a standardized effect of 0.12, 95% CI, 0.03-0.21.

© 2021 Strong DR et al. *JAMA Network Open.*

## eTable 4. Model of Change in Weekly Quitting Cognitions

| | Estimate | Std. Error | | t-value | p-value |
|---|---|---|---|---|---|
| **Age** | | | | | |
| **Age < 45** | -- | -- | | -- | -- |
| **Age 45+** | 0.04 | 0.05 | | 0.77 | 0.45 |
| **Gender** | | | | | |
| **Female** | -- | -- | | -- | -- |
| **Male** | -0.03 | 0.05 | | -0.64 | 0.52 |
| **Nicotine Dependence** | 0.00 | 0.01 | | 0.39 | 0.70 |
| **Quitting Cognitions Pre-Randomization** | | | | | |
| **Pre-Randomization Level (US Pack)** | -0.05 | 0.07 | | -0.66 | 0.51 |
| **Pre-Randomization Level (GWL Pack)** | -0.04 | 0.07 | | -0.53 | 0.59 |
| **Pre-Randomization Level (Blank Pack)** | -0.03 | 0.07 | | -0.37 | 0.71 |
| **Quitting Cognitions During Intervention** | | | | | |
| **Post-Randomization (US Pack)** | -0.03 | 0.07 | | -0.37 | 0.72 |
| **Post-Randomization (GWL Pack)** | 0.11 | 0.07 | | 1.43 | 0.15 |
| **Post-Randomization (Blank Pack)** | 0.09 | 0.07 | | 1.20 | 0.23 |
| **Change Rate in Quitting Cognitions (US Pack, reference)** | | | | | |
| **Weeks during intervention** | 0.03 | 0.004 | | 6.68 | <0.0001 |
| **Differences in Change Rate in Quitting Cognitions (Pack x Week interaction)** | | | | | |
| **US Pack (Weeks during intervention)** | -- | -- | | -- | -- |
| **GWL Pack (Weeks during intervention)** | 0.02 | 0.01 | | 3.26 | 0.001 |
| **Blank Pack (Weeks during intervention)** | 0.00 | 0.01 | | -0.47 | 0.64 |

Note: Estimate = regression model estimate; Std. Error = Standard Error of model estimate; Models estimate relationships with quitting cognitions formed from questions rated in a 4-point scale and normalized to reflect differences from an average rating during the one month baseline period. Rates of change reflect increments in normalized scores each week during the 12-week intervention period. Mean differences between GWL vs US averages during treatment were divided by the pooled standard deviation for a standardized effect of -0.23, 95% CI, 0.34-0.12.

© 2021 Strong DR et al. *JAMA Network Open.*

**eTable 5. Model of Change in Percent of Participants With at Least One Cigarette Abstinence Period/Week**

| | Estimate | Std. Error | | t-value | p-value |
|---|---|---|---|---|---|
| **Age** | | | | | |
| **Age <45** | -- | -- | | -- | -- |
| **Age 45+** | -1.36 | 0.32 | | -4.27 | <0.0001 |
| **Gender** | | | | | |
| **Female** | -- | -- | | -- | -- |
| **Male** | -0.14 | 0.28 | | -0.51 | 0.61 |
| **Nicotine Dependence** | -0.53 | 0.06 | | -8.25 | < 0.0001 |
| **Proportion Abstinent Pre-Randomization** | | | | | |
| **Pre-Randomization Level (US Pack)** | -0.99 | 0.41 | | -2.41 | <0.0001 |
| **Pre-Randomization Level (GWL Pack)** | -1.16 | 0.25 | | -4.64 | 0.62 |
| **Pre-Randomization Level (Blank Pack)** | -0.20 | 0.41 | | -0.49 | 0.02 |
| **Proportion Abstinent During Intervention** | | | | | |
| **Post-Randomization (US Pack)** | -- | -- | | -- | -- |
| **Post-Randomization (GWL Pack)** | 0.32 | 0.40 | | 0.79 | 0.43 |
| **Post-Randomization (Blank [ack)** | -0.30 | 0.40 | | -0.74 | 0.46 |
| **Change Rate in Abstinence During Intervention (US Pack, reference)** | | | | | |
| **Weeks during intervention** | 0.14 | 0.02 | | 5.68 | <0.0001 |
| **Differences in Change Rate in Abstinence During Intervention (Pack x Week interaction)** | | | | | |
| **US Pack (Weeks during intervention)** | -- | -- | | -- | -- |
| **GWL Pack (Weeks during intervention)** | 0.06* | 0.03 | | 1.64 | 0.10 |
| **Blank Pack (Weeks during intervention)** | 0.09 | 0.03 | | 2.53 | 0.01 |

*Effect size comparing GWL vs US reflected by an adjusted odds ratio (AOR) = 1.06 per Week increase, 95% CI: 0.99, 1.13.

© 2021 Strong DR et al. *JAMA Network Open.*

## eTable 6: Model of Change in Cigarette Consumption Reported During Daily EMAs[a]

| | Estimate | Std. Error | | t-value | p-value |
|---|---|---|---|---|---|
| **Age** | | | | | |
| **Age <45** | -- | -- | | -- | -- |
| **Age 45+** | 0.15 | 0.07 | | 2.08 | 0.04 |
| **Gender** | | | | | |
| **Female** | -- | -- | | -- | -- |
| **Male** | -0.06 | 0.07 | | -0.84 | 0.40 |
| **Nicotine Dependence** | -0.04 | 0.01 | | -2.39 | 0.02 |
| **Positive Reactions Weekly Cigarettes** | | | | | |
| **Pre-Randomization Level (US Pack)** | 0.14 | 0.06 | | 2.34 | 0.02 |
| **Pre-Randomization Level (GWL Pack)** | 0.16 | 0.10 | | 1.65 | 0.10 |
| **Pre-Randomization Level (Blank Pack)** | 0.15 | 0.10 | | 1.56 | 0.12 |
| **Weekly[b] Cigarettes During Intervention** | | | | | |
| **Post-Randomization (US Pack)** | -- | -- | | -- | -- |
| **Post-Randomization (GWL Pack)** | -0.03 | 0.10 | | -0.28 | 0.78 |
| **Post-Randomization (Blank Pack)** | -0.01 | 0.10 | | -0.12 | 0.91 |
| **Change Rate in Weekly Cigarettes (US Pack, reference)** | | | | | |
| **In first 2 months** | -0.07 | 0.01 | | -8.15 | <0.0001 |
| **In 3rd month** | 0.11 | 0.03 | | 4.10 | <0.0001 |
| **Differences in Change Rate in Weekly Cigarettes (Pack x Week interaction)** | | | | | |
| **US Pack (In first 2 months)** | -- | -- | | -- | -- |
| **GWL Pack (In first 2 months)** | 0.00 | 0.01 | | 0.33 | 0.74 |
| **Blank Pack (In first 2 months** | -0.01 | 0.01 | | -0.44 | 0.66 |
| **US Pack (Additional slope from 3rd month)** | -- | -- | | -- | -- |
| **GWL Pack (Additional slope from 3rd month)** | -0.07 | 0.04 | | -1.76 | 0.08 |
| **Blank Pack (Additional slope from 3rd month)** | -0.05 | 0.04 | | -1.37 | 0.17 |

[a]EMA: ecological momentary assessment by interactive texting
[b]Weekly consumption was obtained from daily EMAs by taking the average number of cigarettes reported as consumed during the twice daily four-hour windows surveyed.

© 2021 Strong DR et al. *JAMA Network Open.*
