# Supplementary material for: Effect of Graphic Warning Labels on Cigarette Packs on US Smokers’ Cognitions and Smoking Behavior After 3 Months: A Randomized Clinical Trial
Source: JAMA Netw Open. 2021 Aug 4;4(8):e2121387. doi: 10.1001/jamanetworkopen.2021.21387 (PMC8339936; doi:10.1001/jamanetworkopen.2021.21387)
Supplement: Supplement 3. — Data Sharing Statement [file jamanetwopen-e2121387-s003.pdf]

Data Sharing Statement

Strong. Effect of Graphic Warning Labels on Cigarette Packs on US Smokers' Cognitions and Smoking Behavior After 3 Months. *JAMA Netw Open*. Published August 04, 2021.
doi:10.1001/jamanetworkopen.2021.21387

Data
Data available: Yes
Data types: Deidentified participant data
How to access data: send request to jppierce@ucsd.edu
When available: With publication

Supporting Documents
Document types: None

Additional Information
Who can access the data: researchers whose proposed use of the data has been approved
Types of analyses: all analyses included in the paper
Mechanisms of data availability: we plan to put the data up on a website on completion of all study papers. Prior to that, we will need a signed data access agreement
